# Delabeling Antibiotic Allergy in the Solid Organ Transplant Population Using a Multiple Antibiotic Allergy Evaluation Strategy

**DOI:** 10.1111/tid.70099

**Published:** 2025-09-11

**Authors:** Rebecca Lee, Grace Koo, Matthew S. Krantz, Christine Allocco, Elizabeth J. Phillips, Cosby A. Stone

**Affiliations:** ^1^ Division of Infectious Diseases Department of Medicine Vanderbilt University Medical Center Nashville Tennessee USA; ^2^ Division of Allergy, Pulmonary and Critical Care Medicine, Department of Medicine Vanderbilt University Medical Center Nashville Tennessee USA; ^3^ Department of Pathology, Microbiology, and Immunology Vanderbilt University School of Medicine Nashville Tennessee USA; ^4^ Institute for Immunology & Infectious Diseases Murdoch University Perth Western Australia Australia

**Keywords:** antibiotic allergy label, multiple drug delabeling, transplant

## Abstract

**Background:**

First‐line antibiotics, such as penicillins, cephalosporins, and sulfonamides, are critical for preventing infections in immunocompromised solid organ transplant (SOT) patients. However, many patients are labeled with multiple antibiotic allergies (AALs) prior to transplant, increasing their risk of adverse outcomes. Because these patients often travel long distances and follow complex care plans, minimizing the number of drug allergy clinic (DAC) visits is important to avoid disruption and improve care continuity.

**Methods:**

We conducted a retrospective cohort study of SOT patients evaluated at Vanderbilt University Medical Center outpatient DAC between 2014 and 2024. We assessed the efficacy, feasibility, and efficiency of a multiple antibiotic allergy evaluation strategy (MAAES), where patients with two or more low‐risk AALs underwent consolidated evaluation, testing, and oral challenges, with the goal of delabeling as many as three AALs in a single visit.

**Results:**

Among 184 SOT patients referred for evaluation, the median age was 57 years (IQR 47, 64); 112/184 (61%) were female, and 64/184 (35%) traveled from out‐of‐state. A total of 53 patients (29%) had two or more first‐line AALs. Of these, 49 (93%) had labels successfully removed during their visit: 37 penicillin, 25 cephalosporin, and 24 sulfa allergy labels were delabeled. MAAES reduced the number of required visits to address these AALs by 61%.

**Conclusions:**

MAAES enabled safe, efficient, and consolidated AAL evaluation and removal in SOT patients. In 57% of patients with ≥ 2 first‐line AALs, all were safely delabeled in a single clinic visit, improving care efficiency and antibiotic access.

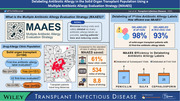

## Graphical abstract

The use of a multiple antibiotic allergy evaluation strategy (MAAES) is an impactful, time‐, and cost‐saving approach to antibiotic allergy testing. MAAES is designed to minimize testing visits and safely delabel as many antibiotic allergy labels, with particular utility for transplant patients.

**Abbreviations**
AAL: antibiotic allergy label
ADR: adverse drug reaction
DAC: drug allergy clinic
EHR: electronic health record
ICD: international classification of diseases
IQR: interquartile range
IRB: Institutional Review Board
MAAES: multiple antibiotic allergy evaluation strategy
MDAL: multiple drug allergy labels
PAL: penicillin allergy label
REDCap: Research electronic data capture software
SOT: solid organ transplantation
VASAP: Vanderbilt Asthma, Sinus, and Allergy Program
VUMC: Vanderbilt University Medical Center

## 1 Introduction

In the general population, first‐line antibiotics like penicillins, cephalosporins, and sulfonamides are often incorrectly labeled as allergies [1] following adverse drug reactions (ADRs) such as nausea, diarrhea, and mild, self‐limited rashes [2]. Many antibiotic allergy labels (AAL) can be removed through appropriate evaluation and testing [3, 4, 5, 6]. Unverified patient‐reported AAL often hinders optimal infection treatment, especially in immunocompromised individuals such as transplant patients [7, 8, 9]. Transplant candidates are particularly susceptible to ADRs due to high‐antibiotic exposure, frequently accumulating multiple unverified allergy labels [10, 11, 12] by the time transplantation is indicated.

Improving access to and the feasibility of drug allergy testing and delabeling is crucial. Currently, patients often travel long distances, see multiple providers, and attend several appointments to address multiple drug allergy labels (MDAL). This fragmented approach contributes to delays in care, patient dissatisfaction, increased costs, and suboptimal health outcomes [13].

We previously demonstrated that evaluating multiple drug allergies in a single visit is feasible, successfully testing and delabeling more than one drug in 461 of 536 (86%) patients. However, a review 1 year later revealed that 134 of these patients (25%) were relabeled with at least one previously removed allergy [13].

To address logistical challenges, the Vanderbilt Drug Allergy Clinic created a multiple antibiotic allergy evaluation strategy (MAAES) to reduce testing visits and enhance communication with patients' pharmacies and all outside providers. We aimed to evaluate the efficacy, feasibility, and efficiency of testing and delabeling MDAL in solid organ transplant (SOT) patients during their first clinic visit, assessing the effectiveness of MAAES in saving time and reducing costs.

## 2 Methods

### 2.1 Study Design

We conducted a retrospective cohort study of 184 adult SOT patients at the Vanderbilt Drug Allergy Clinic at Vanderbilt University Medical Center (VUMC) from 2014 to 2024. We assessed the efficacy of single and multiple antibiotic allergy delabeling using MAAES, wherein patients with low‐risk AALs underwent evaluation, testing, and challenges in one visit. Our study examined MAAES efficiency for patients with two or more first‐line AALs delabeled in a single visit.

We abstracted, recorded, and analyzed data using REDCap, a secure, HIPAA‐compliant research electronic data capture system. We used the VUMC Drug Allergy Clinic Database (IRB #161455), created on July 16, 2019, as the primary data source for patient data. We conducted retrospective chart reviews to obtain patient information, including demographics, state of residence, transplant type, and the antibiotics tested and delabeled in a single visit.

### 2.2 Patient Characteristics

**FIGURE 1:**
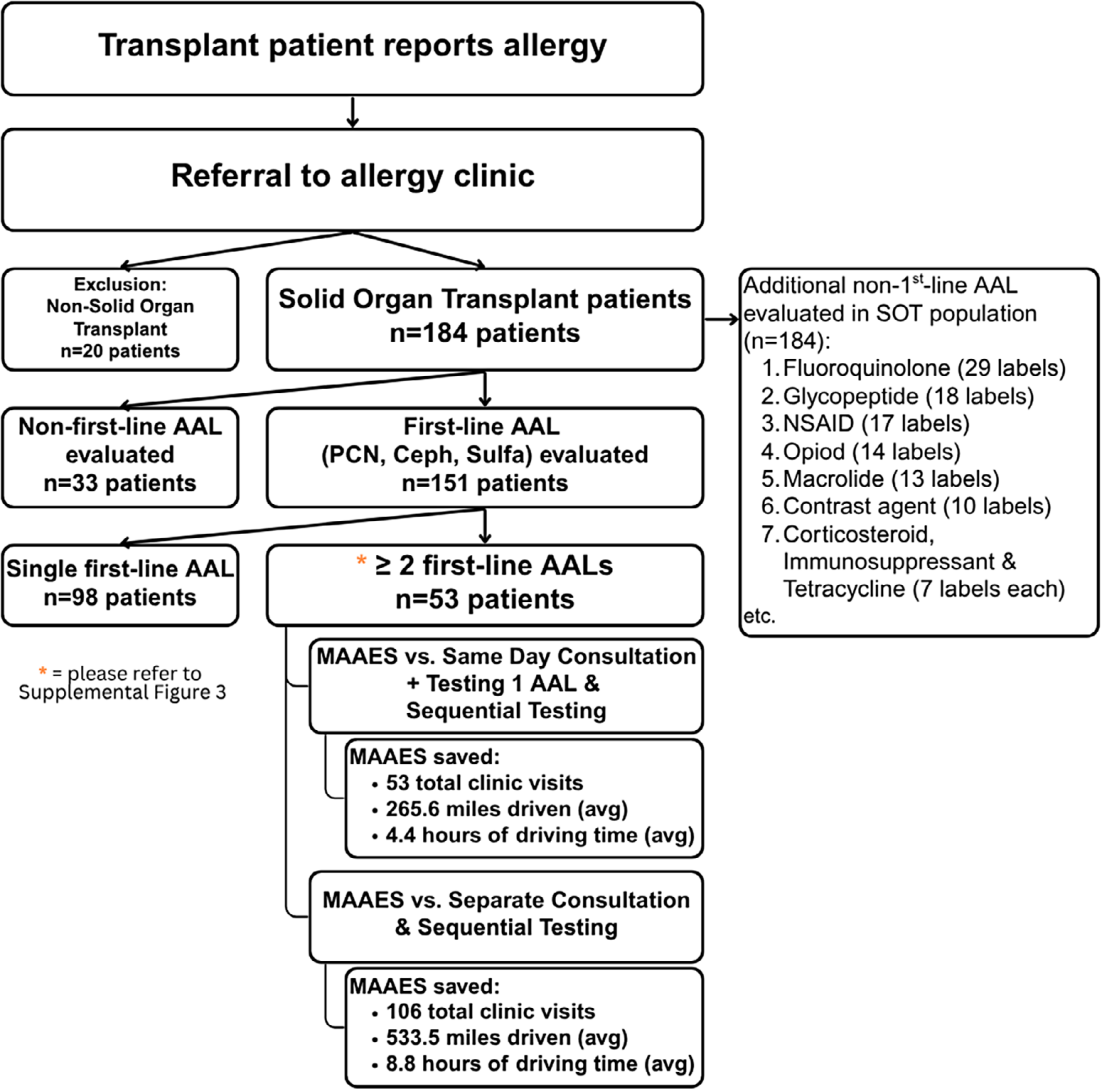
Transplant Population Consort diagram.

From over 3000 patients seen at the VUMC drug allergy clinic, we included all patients with an SOT listed on the ICD‐10 codes in the Electronic Health Record (EHR) who were evaluated for drug allergies before or after their transplant. Non‐SOT (*n* = 20) patients were excluded (Figure 1).

### 2.3 Innovation: Multiple Antibiotic Allergy Evaluation Strategy (MAAES)

This study focuses on AALs to penicillins, cephalosporins, and sulfonamides in transplant patients, guided by validated protocols [3, 4, 5, 6, 14, 15, 16, 17]. Given the long distances, many patients must travel for care and the complex referral patterns, we developed a one‐day MAAES protocol to efficiently test MDALs and delabel as many as possible without requiring return visits. This process had been conceived since the clinic's inception in March 2014 and came into existence due to the growing recognition that the exceeding majority of AAL currently listed in the EHR can be removed when evaluated and tested [13].

Our MAAES protocol targets delabeling remote, low‐risk AAL through skin testing (all indicated drugs) and ingestion challenges (up to three antibiotics) in one visit. Skin testing with penicillins and cephalosporins occurs routinely for higher‐risk patients, per established protocols [3, 4]. Sulfonamides are not routinely tested due to poor predictive value; instead, they are used in either one or two‐step direct challenges [5, 6]. Sequential one‐day oral challenges enable beta‐lactam drug tests (with a short half‐life) to be conducted with a 1‐ to 1.5‐h observation period. The final challenge with trimethoprim‐sulfamethoxazole requires at least 2 h due to its longer half‐life. These innovations enable delabeling multiple antibiotics in a 4–6‐hour visit. Since 2020, additional evidence led us to include sequential direct challenges, skipping skin tests for low‐risk index reactions when possible [4, 5, 6, 15, 18, 19]. We monitor patients with vital sign measurements and elicitation of any symptoms every 30 min during oral challenges and with follow‐up phone calls or secure EHR messages 24 h posttest.

### 2.4 Comparing Cost and Time Savings in Different Testing Strategies

**FIGURE 2:**
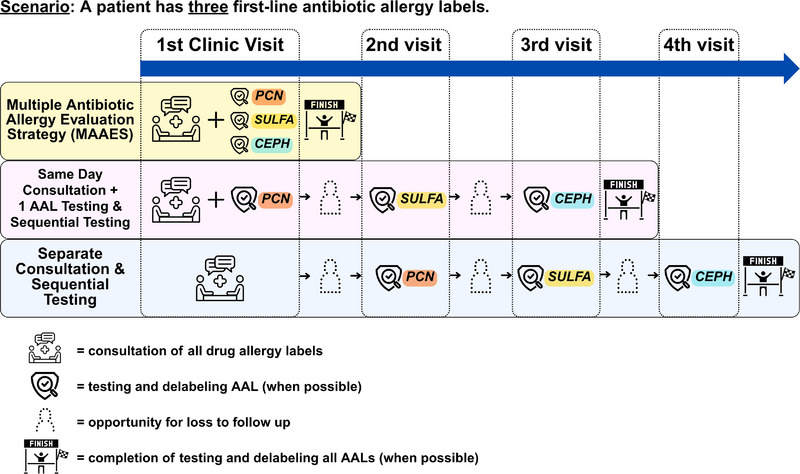
Comparison between MAAES and other sequential testing strategies. Scenario: A patient has three first‐line AALs.

The standard care in allergy clinics is to evaluate one AAL per visit. Most recommend a “same‐day approach” to consult all AALs and test one on the first visit, assessing others sequentially. Alternatively, “separate” consultation and evaluation of each AAL are in distinct visits, creating inefficiencies and burdens, especially for immunocompromised SOT patients. In MAAES, providers aim to consolidate consultation and all AAL testing into one visit, offering a more efficient evaluation strategy (Figure 2).

For patients with MDAL, strategies other than MAAES can result in numerous appointments, which are time‐consuming and lack feasibility for the patient [13]. We evaluated the driving distance and time between each transplant patient's residential zip code and our clinic to compare the time and cost savings between MAAES and two standard approaches to sequential allergy label evaluation.

## 3 Results

### 3.1 Patient Demographics and Transplant Types

Among 204 transplant patients at VUMC drug allergy clinic from 2014 to 2024, 184 were SOT candidates or recipients as part of a pretransplant or posttransplant workup of relevant drug allergy labels. Thirteen had received more than one type of SOT. The median age was 58 years (IQR 47, 65); 61% were female, and 64/184 (35%) lived out‐of‐state, representing 11 states across the Southeast and Midwest. Lung transplants accounted for the largest subgroup (51.6%) (Table S1), reflecting an increase in lung transplants since 2019 and consistent referral patterns to the clinic.

### 3.2 Efficacy: Testing and Delabeling Antibiotic Allergy With MAAES

Among 184 SOT patients, 151 (82.1%) were evaluated for first‐line AAL. The remaining 33 were evaluated for non‐first‐line antibiotic allergies, including fluoroquinolones and glycopeptides. Of the 151, 138 (91.4%) had these labels successfully removed across all visits, and 133 (88.1%) were delabeled at the initial visit (Table S2).

Within this cohort, 53 patients (35.1%) had ≥ 2 AALs: 39 had two AALs, and 14 had three (Table S2). Of these 49 (92.5%), multiple AALs were evaluated and delabeled during their first visit. Specifically, 37 of 44 (84.1%) penicillin AALs, 24 of 33 (72.7%) sulfa AALs, and 25 of 43 (58.1%) cephalosporin AALs were delabeled at the first visit (Figure S1), with prioritization of penicillin and sulfa evaluation. No safety concerns leading to relabeling of an allergy were reported in these patients during their testing day or follow‐up phone calls.

MAAES evaluated 53 MDAL patients aiming to delabel all first‐line AALs. Of these, 37 (70%) completed all evaluations during their first visit: 30 were fully delabeled (ideal), 5 had one true allergy with others delabeled, and 2 had modified treatment regimens (like premedicating with antihistamines) plus other drug delabeling, demonstrating the efficacy, feasibility, and efficiency of MAAES. The remaining 16 (30%) could not finish evaluations in one visit due to time constraints from other transplant‐related appointments. Ten returned for follow‐up and had all first‐line AALs delabeled on the second visit, except one who requested a third. Six were lost to follow‐up. The primary reasons for returns and lost follow‐ups included patients' time constraints and disinclination to further testing. We utilize MAAES for all MDAL patients when appropriate, but sometimes face less efficient outcomes due to patient requests.

Among 64 out‐of‐state SOT patients, 14 were assessed for ≥ 2 first‐line AALs. Of these, 11 (78.6%) successfully had all AALs delabeled at their initial visit, demonstrating MAAES's role in reducing the travel burden and consolidating care.

### 3.3 Effective Outcomes: Time and Mileage Savings

We compared MAAES with two conventional strategies—“separate” and “same‐day” sequential evaluations among 53 SOT patients with ≥ 2 first‐line AALs. Based on the number of AALs evaluated, standard approaches would have required 173 (“separate”) or 120 (“same‐day”) visits. MAAES reduced this to 67 visits, resulting in a 61.3% and 44.2% reduction, respectively. Of the 67 total MAAES visits, only 15 were return appointments (Table S3).

Travel data further illustrates the efficiency of MAAES. Patients using a “separate” approach would have driven a theoretical average of 850 miles for all required visits, compared to 582 miles for the “same‐day” approach. In contrast, MAAES patients average only 316 miles, saving hundreds of miles per patient (Table S3).

Estimated travel time also decreased proportionately and significantly. Without MAAES, average total round‐trip times would have been 14 h (“separate”) and 9.5 h (“same‐day”). MAAES reduced this to 5.1 h, reflecting substantial reductions in travel time, patient burden, and costs (Table S3).

### 3.4 Beyond First‐Line Antibiotic Allergy Labels

Vanderbilt Asthma, Sinus, and Allergy Program (VASAP) providers also evaluated and delabeled non‐first‐line AALs, providing essential educational advice on drug safety and patient allergy awareness. They review each patient's medical history to identify other allergy risks. Among the 184 SOT population, 111 (60.3%) had additional non‐first‐line allergy labels, totaling 206. The most common labels were fluoroquinolones (14%) and glycopeptides (8.7%) (Table S4).

## 4 Discussion

Our study is the first to evaluate MAAES against two standard sequential evaluation strategies. All strategies aim to delabel unnecessary AALs; however, MAAES demonstrated efficacy, feasibility, and efficiency, with 75% of SOT and 70% of MDAL patients having all first‐line AALs consulted, tested, and delabeled during a single visit. Furthermore, 93% of MDAL patients had at least one first‐line AAL delabeled during all visits, while 98% were delabeled on their first visit (Figure S1).

In a scenario of having many patients who need multiple tests for multiple allergies, reducing return visits is a key element of resource stewardship, because it frees up appointment slots for other patients and enables earlier care, creating a virtuous cycle. We observed that the MAAES approach saved MDAL patients from 106 additional return visits across 53 patients, reducing travel expenses and driving time.

While we observed that six patients had incomplete evaluations and were lost to follow‐up, Figure 2 shows a higher potential for loss to follow‐up among MDAL patients in the other two evaluation approaches, highlighting the burden of multiple visits. Over time, less‐efficient approaches may lead to reduced compliance and increased time, cost, and energy burdens, resulting in persistent, untested AALs in EHR and adverse health outcomes. The main barrier to completing all testing in one day with the MAAES approach is patient fatigue and limited time, as transplant‐related appointments often require early departure. Cumulative oral challenge fatigue may lead some patients to prefer spreading challenges over multiple days. However, once set up to be a clinic that can perform a MAAES workflow, there is no additional incremental cost to operating in this way, since the presence of key staff and clinic space during operating hours is a sunk cost. Reimbursement for in‐office challenges is also based on observation time, which remains the same whether it is provided across one day or multiple days. Therefore, we offer and use a MAAES approach for all MDAL patients, but occasionally face less‐efficient testing outcomes when accommodating individual patient‐centered needs.

First‐line antibiotics are vital for SOT patients to prevent and treat posttransplant infections. Reported allergy labels in EHRs may lead to using less effective, costlier alternative antibiotics, potentially worsening health outcomes [20, 21, 22]. Delabeling sulfonamide antibiotics in pretransplant patients saved $254–$2910 per patient every 6 months, totaling $3244–$32 460 for 11 patients compared to non‐first‐line sulfonamide antibiotics [23]. Further, for health outcomes, a previous 2‐year study revealed penicillin allergy label (PAL) patients had 5.5% longer hospital stays than non‐PAL patients, with 3522 excess bed‐days [24]. Preventing longer hospital stays through advanced delabeling may save more in‐future costs than alternative antibiotics. Thus, we should delabel transplant patients of AALs when feasible, to use effective, cost‐efficient first‐line antibiotics and reduce future healthcare burdens. Our study is key to defining less burdensome delabeling methods for already‐strained transplant patients.

A key limitation in our current report is that we have examined travel, visit, and time savings, but have not assessed all potential costs. Multiple DAC appointments may incur extra co‐pays, costly hotel stays in Nashville, lost work hours, and delays in seeing patients due to limited appointment availability. These additional unassessed costs and the burden of travel can adversely affect SOT patients' finances, time, other health expenses, and mental health [25]. While we haven't yet calculated the actual monetary savings that may be present in our approach, we have focused on the key cost drivers of time involved in clinic visits, mileage, and driving hours in our patient population.

## 5 Conclusion

In conclusion, the implementation of MAAES was efficacious in delabeling all AALs from the majority of MDAL patients in a single visit, thereby effectively saving patients from additional visits, travel time, and associated costs. Utilizing a MAAES strategy can also improve DAC providers' output, allowing them to dedicate more visits to new patients and increase overall accessibility.

## Ethics Statement

This Study Followed IRB‐approved Protocols from Vanderbilt University, IRB #161455.

## Conflicts of Interest

The authors declare no conflicts of interest.

## Supporting information




**Supporting Fig 1**: Antibiotic allergy label (AAL) priority in delabeling MDAL patients.

**Supporting Table 1**: Demographics of Solid‐Organ Transplant (SOT) patient population seen for drug allergy.

**Supporting Table 2**: Delabeling in Solid Organ Transplant Patients.

**Supporting Table 3**: Comparison between MAAES and other sequential testing strategies.

**Supporting Table 4**: Non‐AAL evaluations.

**Supporting File 1**: tid70099‐sup‐0006‐VisualAbstract.png

## References

[1] C. A. Stone , J. Trubiano , D. T. Coleman , C. R. F. Rukasin , and E. J. Phillips , “The Challenge of De‐Labeling Penicillin Allergy,” Allergy 75, no. 2 (2020): 273–288. 31049971 10.1111/all.13848 PMC6824919

[2] J. A. Trubiano , N. F. Adkinson , and E. J. Phillips , “Penicillin Allergy Is Not Necessarily Forever,” JAMA 318, no. 1 (2017): 82. 28672303 10.1001/jama.2017.6510 PMC5935455

[3] C. A. Stone , J. A. Trubiano , and E. J. Phillips , “Testing Strategies and Predictors for Evaluating Immediate and Delayed Reactions to Cephalosporins,” Journal of Allergy and Clinical Immunology: In Practice 9, no. 1 (2021): 435–444.e13. 32822918 10.1016/j.jaip.2020.07.056 PMC7855229

[4] G. Koo , R. Yu , E. J. Phillips , and C. A. Stone Jr , “Retrospective Stratification of Cephalosporin Allergy Label Risk Using Validated Penicillin Allergy Frameworks,” Journal of Allergy and Clinical Immunology: In Practice 10, no. 9 (2022): 2472–2475. 35690369 10.1016/j.jaip.2022.05.032 PMC13126447

[5] M. S. Krantz , C. A. Stone , A. Abreo , and E. J. Phillips , “Reply to “The Safety and Efficacy of Direct Oral Challenge in Trimethoprim‐Sulfamethoxazole Antibiotic Allergy”,” Journal of Allergy and Clinical Immunology: In Practice 9, no. 10 (2021): 3849–3850. 34627544 10.1016/j.jaip.2021.07.027 PMC12709610

[6] M. S. Krantz , C. A. Stone , A. Abreo , and E. J. Phillips , “Oral Challenge With Trimethoprim‐Sulfamethoxazole in Patients With “Sulfa” Antibiotic Allergy,” Journal of Allergy and Clinical Immunology: In Practice 8, no. 2 (2020): 757–760.e4. 31319222 10.1016/j.jaip.2019.07.003 PMC6960372

[7] L. M. Abbo and E. J. Ariza‐Heredia , “Antimicrobial Stewardship in Immunocompromised Hosts,” Infectious Disease Clinics of North America 28, no. 2 (2014): 263–279. 24857392 10.1016/j.idc.2014.01.008

[8] J. A. Trubiano , K. A. Thursky , A. J. Stewardson , et al., “Impact of an Integrated Antibiotic Allergy Testing Program on Antimicrobial Stewardship: A Multicenter Evaluation,” Clinical Infectious Diseases 65, no. 1 (2017): 166–174. 28520865 10.1093/cid/cix244 PMC5849110

[9] J. A. Trubiano , M. L. Grayson , K. A. Thursky , E. J. Phillips , and M. A. Slavin , “How Antibiotic Allergy Labels May Be Harming Our Most Vulnerable Patients,” Medical Journal of Australia 208, no. 11 (2018): 469–470. 29902399 10.5694/mja17.00487 PMC6167469

[10] H. Imlay , E. M. Krantz , E. J. Stohs , et al., “Reported β‐Lactam and Other Antibiotic Allergies in Solid Organ and Hematopoietic Cell Transplant Recipients,” Clinical Infectious Diseases 71, no. 7 (2019): 1587–1594. 10.1093/cid/ciz1025 PMC8241219 31621829

[11] A. Guyer , M. Iammatteo , M. Karagic , E. Macy , and E. Jerschow , “Tackling the Patient With Multiple Drug “Allergies”: Multiple Drug Intolerance Syndrome,” Journal of Allergy and Clinical Immunology: In Practice 8, no. 9 (2020): 2870–2876. 33039011 10.1016/j.jaip.2020.08.033

[12] J. A. Trubiano , M. A. Slavin , K. A. Thursky , M. L. Grayson , and E. J. Phillips , “Beta‐Lactam and Sulfonamide Allergy Testing Should Be a Standard of Care in Immunocompromised Hosts,” Journal of Allergy and Clinical Immunology: In Practice 7, no. 7 (2019): 2151–2153. 31253580 10.1016/j.jaip.2019.05.051 PMC6733665

[13] C. Vethody , R. Yu , J. M. Keck , M. K. Onasch , C. A. Stone , and E. J. Phillips , “Safety, Efficacy, and Effectiveness of Delabeling in Patients With Multiple Drug Allergy Labels,” Journal of Allergy and Clinical Immunology: In Practice 9, no. 2 (2021): 922–928. 32966878 10.1016/j.jaip.2020.09.010 PMC8187885

[14] M. Staub , G. E. Nelson , K. Byrge , et al., “Impacts of Risk‐Stratified Inpatient Penicillin Allergy Label Delabeling on Subsequent Antimicrobial Spectrum Index and Costs,” Antimicrobial Stewardship & Healthcare Epidemiology 4, no. 1 (2024): e160. 39371443 10.1017/ash.2024.421 PMC11450671

[15] G. Koo , J. L. Stollings , C. Lindsell , et al., “Low‐Risk Penicillin Allergy Delabeling Through a Direct Oral Challenge in Immunocompromised and/or Multiple Drug Allergy Labeled Patients in a Critical Care Setting,” Journal of Allergy and Clinical Immunology: In Practice 10, no. 6 (2022): 1660–1663.e2. 35131513 10.1016/j.jaip.2022.01.041 PMC9188986

[16] C. A. Stone , J. L. Stollings , C. J. Lindsell , et al., “Risk‐Stratified Management to Remove Low‐Risk Penicillin Allergy Labels in the ICU,” American Journal of Respiratory and Critical Care Medicine 201, no. 12 (2020): 1572–1575. 32083961 10.1164/rccm.202001-0089LE PMC7301733

[17] J. A. Trubiano , S. Vogrin , K. Y. L. Chua , et al., “Development and Validation of a Penicillin Allergy Clinical Decision Rule,” JAMA Internal Medicine 180, no. 5 (2020): 745. 32176248 10.1001/jamainternmed.2020.0403 PMC7076536

[18] J. Moraczewski , R. Lee , G. Koo , et al., “Direct Oral Challenges Safely Reduce the Burden of Low‐Risk Cephalosporin Allergy Labels,” Journal of Allergy and Clinical Immunology: In Practice, ahead of print, 2025: S2213‐2198(25)00531‐8. 10.1016/j.jaip.2025.05.054 PMC12606405 40484351

[19] A. M. Copaescu , S. Vogrin , F. James , et al., “Efficacy of a Clinical Decision Rule to Enable Direct Oral Challenge in Patients With Low‐Risk Penicillin Allergy,” JAMA Internal Medicine 183, no. 9 (2023): 944. 37459086 10.1001/jamainternmed.2023.2986 PMC10352926

[20] E. Macy and R. Contreras , “Health Care Use and Serious Infection Prevalence Associated With Penicillin “Allergy” in Hospitalized Patients: A Cohort Study,” Journal of Allergy and Clinical Immunology 133, no. 3 (2014): 790–796. 24188976 10.1016/j.jaci.2013.09.021

[21] Y. Y. Ham , S. Joshi , and E. Sukerman , “Delabeling Penicillin and Other Antibiotic Allergies in Solid Organ Transplantation Patients,” Transplant Infectious Disease 24, no. 5 (2022): e13897. 36254514 10.1111/tid.13897

[22] J. Trubiano and E. Phillips , “Antimicrobial Stewardship's New Weapon? A Review of Antibiotic Allergy and Pathways to ‘De‐Labeling’,” Current Opinion in Infectious Diseases 26, no. 6 (2013): 526–537. 24126717 10.1097/QCO.0000000000000006 PMC3862073

[23] C. A. Gorsline , A. K. Afghan , C. A. Stone , E. J. Phillips , and G. Satyanarayana , “Safety and Value of Pretransplant Antibiotic Allergy Delabeling in a Quaternary Transplant Center,” Transplant Infectious Disease 24, no. 5 (2022): e13885. 35765165 10.1111/tid.13885 PMC9588656

[24] N. Powell , K. Honeyford , and J. Sandoe , “Impact of Penicillin Allergy Records on Antibiotic Costs and Length of Hospital Stay: A Single‐Centre Observational Retrospective Cohort,” Journal of Hospital Infection 106, no. 1 (2020): 35–42. 32502582 10.1016/j.jhin.2020.05.042

[25] P. H. Li and B. Y.‐H. Thong , “Delabelling Multiple Antibiotic Allergy: Practical Issues,” Frontiers in Allergy 4 (2023): 1156137. 37007647 10.3389/falgy.2023.1156137 PMC10061016

