# Supplementary figures and images for: Delabeling Antibiotic Allergy in the Solid Organ Transplant Population Using a Multiple Antibiotic Allergy Evaluation Strategy
Source: Transpl Infect Dis. 2025 Sep 11;27(5):e70099. doi: 10.1111/tid.70099 (PMC12519911; doi:10.1111/tid.70099)

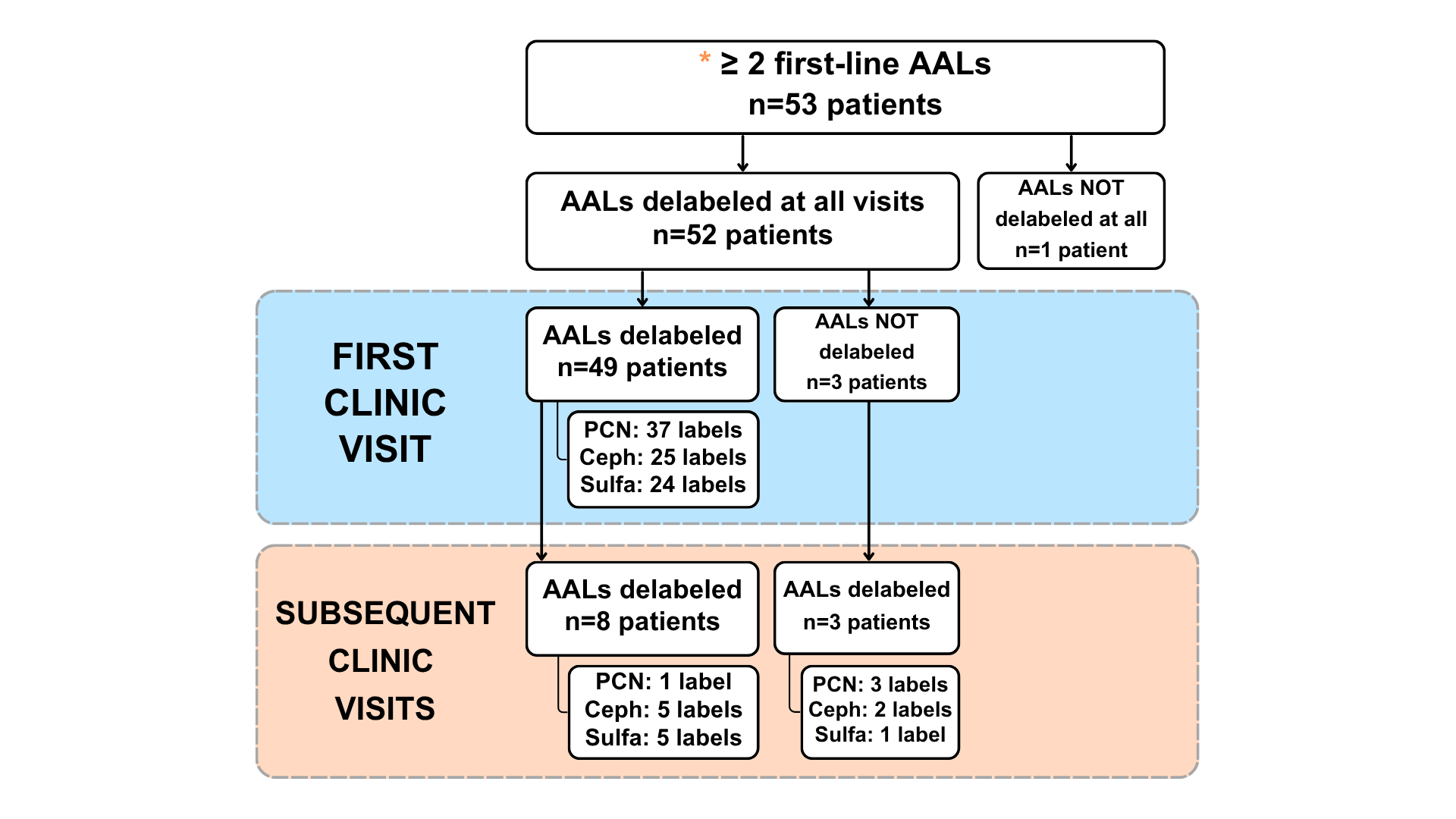

Supplement: Supplementary file 1 — Supporting Fig 1: Antibiotic allergy label (AAL) priority in delabeling MDAL patients. [file TID-27-e70099-s003.png]

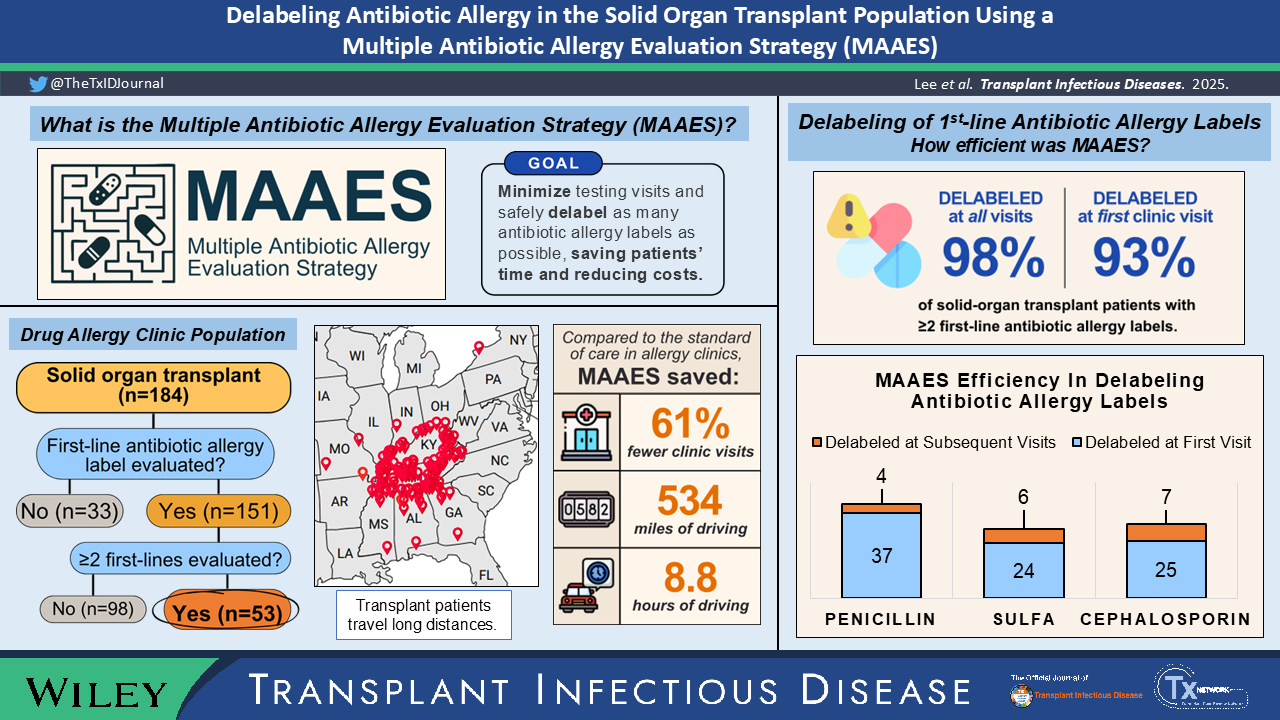

Supplement: Supplementary file 6 — Supporting File 1: tid70099‐sup‐0006‐VisualAbstract.png [file TID-27-e70099-s005.png]
