# Supplementary material for: Delabeling Antibiotic Allergy in the Solid Organ Transplant Population Using a Multiple Antibiotic Allergy Evaluation Strategy
Source: Transpl Infect Dis. 2025 Sep 11;27(5):e70099. doi: 10.1111/tid.70099 (PMC12519911; doi:10.1111/tid.70099)
Supplement: Supplementary file 2 — Supporting Table 1: Demographics of Solid‐Organ Transplant (SOT) patient population seen for drug allergy. [file TID-27-e70099-s002.docx]

**Supplemental Table 1. Demographics of Solid-Organ Transplant (SOT) patient population seen for drug allergy**

|  | **Characteristics** | **All patients (n=184)** | **Patients with ≥2 1st-line AALs (n=53)** |
| --- | --- | --- | --- |
| **Age (yrs)** | Median (IQR) | 57.7 [47.6 - 64.7] | 56.8 [49.6 - 66] |
| **Sex** | Female | 112 (60.9) | 39 (73.6) |
|  | Male | 72 (39.1) | 14 (26.4) |
| **Self-Reported Race** | American Indian/Alaska Native | 0 (0) | 0 (0) |
|  | Asian | 2 (1.09) | 0 (0) |
|  | Native Hawaiian or Other Pacific Islander | 0 (0) | 0 (0) |
|  | Black or African American | 24 (13.04) | 5 (9.4) |
|  | White | 152 (82.61) | 48 (90.6) |
|  | Other Race | 0 (0) | 0 (0) |
|  | Unknown | 6 (3.26) | 0 (0) |
| **Ethnicity** | Hispanic or Latino | 4 (2.17) | 1 (1.9) |
|  | Not Hispanic or Latino | 172 (93.48) | 51 (96.2) |
|  | Unknown | 8 (4.35) | 1 (1.9) |
| **Transplant** | Lung | 95 (51.63) | 21 (39.6) |
|  | Liver | 12 (6.52) | 6 (11.3) |
|  | Heart | 45 (24.46) | 12 (22.6) |
|  | Kidney | 18 (9.78) | 9 (17) |
|  | Pancreas | 1 (0.54) | 1 (1.9) |
|  | Multiple  Heart & liver  Heart & stem cell  Kidney & heart  Kidney & liver  Kidney & lung  Kidney & pancreas | 13 (7.07)  2  1  6  2  1  1 | 4 (7.6)  0  0  2  1  0  1 |
| **State of Residence** | In-State | 120 (65.22) | 39 (73.6) |
|  | Out-of-State | 64 (34.78) | 14 (26.4) |
