# Supplementary material for: Delabeling Antibiotic Allergy in the Solid Organ Transplant Population Using a Multiple Antibiotic Allergy Evaluation Strategy
Source: Transpl Infect Dis. 2025 Sep 11;27(5):e70099. doi: 10.1111/tid.70099 (PMC12519911; doi:10.1111/tid.70099)
Supplement: Supplementary file 3 — Supporting Table 2: Delabeling in Solid Organ Transplant Patients. [file TID-27-e70099-s004.docx]

**Supplemental Table 2. Delabeling in Solid Organ Transplant Patients**

|  | **Patients Evaluated (n=184)** | **Patients Delabeled at All Visits** | **Patients Delabeled at 1st Clinic Visit** |
| --- | --- | --- | --- |
| **1st-line AALs Evaluated and Delabeled** | 0 antibiotic (n=33) |  |  |
|  | 1 antibiotic (n=98) | 86 | 84 |
|  | 2 antibiotics (n=39)  Delabeled only 1 antibiotic  Delabeled all 2 antibiotics | 38  8  30 | 36  11  25 |
|  | 3 antibiotics (n=14)  Delabeled only 1 antibiotic  Delabeled only 2 antibiotics  Delabeled all 3 antibiotics | 14  1  5  8 | 13  4  6  3 |
