# Supplementary material for: Delabeling Antibiotic Allergy in the Solid Organ Transplant Population Using a Multiple Antibiotic Allergy Evaluation Strategy
Source: Transpl Infect Dis. 2025 Sep 11;27(5):e70099. doi: 10.1111/tid.70099 (PMC12519911; doi:10.1111/tid.70099)
Supplement: Supplementary file 4 — Supporting Table 3: Comparison between MAAES and other sequential testing strategies. [file TID-27-e70099-s006.docx]

**Supplemental Table 3. Comparison of 1) Separate Consultation & Sequential Evaluation; 2) Same Day Consultation + 1 AAL Testing & Sequential Evaluation; and 3) MAAES, focusing on time and cost savings for MDAL patients**

|  | **Number of clinic visits** | | | **Average miles driven** | | | **Average driving time (hours)** | | |
| --- | --- | --- | --- | --- | --- | --- | --- | --- | --- |
|  | †  Separate Sequential Testing | ‡  Same Day Sequential Evaluation | MAAES | †  Separate Sequential Testing | ‡  Same Day Sequential Evaluation | MAAES | †  Separate Sequential Testing | ‡  Same Day Sequential Evaluation | MAAES |
| Lung  (n=21) | 69 | 48 | 28 | 906.3 | 621.9 | 357.8 | 14.8 | 10.2 | 5.8 |
| Liver  (n=6) | 20 | 14 | 7 | 606.3 | 426.3 | 209.3 | 10.9 | 7.6 | 3.7 |
| Heart (n=12) | 40 | 28 | 16 | 982 | 680 | 366 | 15.6 | 10.8 | 5.8 |
| Kidney (n=9) | 28 | 19 | 10 | 644.4 | 433.3 | 234.4 | 10.8 | 7.2 | 3.9 |
| §Pancreas (n=1) | 3 | 2 | 1 | 918 | 612 | 306 | 14.2 | 9.5 | 4.7 |
| Multiple (n=4) | 13 | 9 | 5 | 1041.5 | 717.5 | 424 | 16.8 | 11.6 | 6.8 |

† = Separate: consultation of all antibiotic allergy labels (AALs) would count as one clinic visit, and each AAL would be tested per visit.

‡ = Same day: consultation of all antibiotic allergy labels (AALs) and testing of 1 antibiotic would be done same day, at the first clinic visit. The remaining AALs would be tested on a per-visit basis.

§ = Single value. Only one patient with a pancreas transplant was seen at our drug allergy clinic.
