# Supplementary material for: Delabeling Antibiotic Allergy in the Solid Organ Transplant Population Using a Multiple Antibiotic Allergy Evaluation Strategy
Source: Transpl Infect Dis. 2025 Sep 11;27(5):e70099. doi: 10.1111/tid.70099 (PMC12519911; doi:10.1111/tid.70099)
Supplement: Supplementary file 5 — Supporting Table 4: Non‐AAL evaluations. [file TID-27-e70099-s001.docx]

**Supplemental Table 4. Additional allergy labels evaluated among 184 SOT patients at VASAP**

| **Non-1^st^-line AALs** | **Number of LABELS (206 total)** |
| --- | --- |
| Fluoroquinolone | 29 |
| Glycopeptide | 18 |
| NSAID | 17 |
| Opioid | 14 |
| Macrolide | 13 |
| Contrast agent | 10 |
| Corticosteroid | 7 |
| Immunosuppressant | 7 |
| Tetracycline | 7 |
| Anticoagulant | 6 |
| Antifungal | 6 |
| Antiseptic | 6 |
| Statin | 5 |
| Calcium channel blocker | 4 |
| Neuromuscular blocker | 4 |
| Aminoglycoside | 3 |
| Anesthetic | 3 |
| Antidepressant | 3 |
| Carbapenem | 3 |
| Local anesthetic | 3 |
| Vaccine | 3 |
| ACE inhibitor | 2 |
| Biguanide | 2 |
| Lincosamide | 2 |
| Nitroimidazole | 2 |
| Oxazolidinone | 2 |
| Analgesic | 1 |
| Anticonvulsant | 1 |
| Antiemetic | 1 |
| Antipsychotic | 1 |
| Antiviral | 1 |
| ARB | 1 |
| Benzodiazepine | 1 |
| Beta blocker | 1 |
| Catecholamine | 1 |
| Diuretic | 1 |
| Endothelin receptor antagonist | 1 |
| Gelatin | 1 |
| Iron | 1 |
| LTRA | 1 |
| Monobactam | 1 |
| Monoclonal antibody | 1 |
| Nasal decongestant | 1 |
| Ophthalmic lubricant | 1 |
| Photosensitivity | 1 |
| PPI | 1 |
| SGLT2 inhibitor | 1 |
| SSRI | 1 |
| Steroid | 1 |
| Sulfone | 1 |
| Xanthine oxidase inhibitor | 1 |
